# Midterm Results of Microhook ab Interno Trabeculotomy in Initial 560 Eyes with Glaucoma

**DOI:** 10.3390/jcm10040814

**Published:** 2021-02-17

**Authors:** Masaki Tanito, Kazunobu Sugihara, Aika Tsutsui, Katsunori Hara, Kaoru Manabe, Yotaro Matsuoka

**Affiliations:** 1 Department of Ophthalmology, Faculty of Medicine, Shimane University, Izumo 693-8501, Japan; ksugi@med.shimane-u.ac.jp (K.S.); aika0408@med.shimane-u.ac.jp (A.T.); hakari55@med.shimane-u.ac.jp (K.H.); 2 Division of Ophthalmology, Matsue Red Cross Hospital, Matsue 690-8506, Japan; manabe42@med.shimane-u.ac.jp (K.M.); ymatsu@med.shimane-u.ac.jp (Y.M.)

**Keywords:** minimally invasive glaucoma surgery (MIGS), Tanito microhook (TMH), surgical efficacy, surgical complication

## Abstract

All the 560 glaucomatous eyes of 375 Japanese subjects (181 men, 194 women; mean age ± standard deviation, 76.0 ± 13.2 years) who underwent microhook ab interno trabeculotomy (µLOT) alone (159 eyes, 28%) or combined µLOT and cataract surgery (401 eyes, 72%) performed by one surgeon at Matsue Red Cross Hospital between May 2015 and March 2018 to control intraocular pressure (IOP) were retrospectively assessed. Preoperative and postoperative IOPs, numbers of antiglaucoma medications, the logarithm of the minimum angle of resolution visual acuity (logMAR VA), anterior chamber (AC) flare, visual field mean deviation (MD), and corneal endothelial cell density (CECD) were compared up to 36 months. Surgical complications and required interventions were described. The duration of the follow-up was 405 ± 327 (range, 2–1326) days. The mean preoperative IOP (20.2 ± 7.0 mmHg) and number of antiglaucoma medications (2.8 ± 1.1) decreased to 13.9 ± 4.5 mmHg (31% reduction, *p* < 0.0001) and 2.5 ± 1.0 (11% reduction, *p* < 0.0001), respectively, at the final visit. After combined surgery, compared with preoperatively, the final VA improved 0.11 logMAR (*p* < 0.0001), AC flare increased 4.5 photon counts/msec (*p* = 0.0011), MD improved 0.6 decibel (*p* < 0.0001), and the CECD decreased 6% (*p* < 0.0001). Layered hyphema (172 eyes, 31%) and hyphema washout (26 eyes, 5%) were the most common postoperative complication and intervention, respectively. At the final visit, 379 (69%) eyes achieved successful IOP control of ≤18 mmHg and ≥20% IOP reduction, and 349 (64%) eyes achieved successful IOP control of ≤15 mmHg and ≥20% IOP reduction. Older age, steroid-induced glaucoma, developmental glaucoma, and the absence of postoperative complications were associated with lower final IOP; exfoliation glaucoma, other types of glaucoma, and higher preoperative IOP were associated with higher final IOP. µLOT has a significant IOP-lowering potential in patients with glaucoma, and improves visual function when combined with cataract surgery.

## 1. Introduction

The intraocular pressure (IOP) in adults and children with glaucoma is reduced by trabeculotomy (LOT), which alleviates the resistance to aqueous flow by cleaving the trabecular meshwork (TM) and inner walls of the Schlemm’s canal [1,2,3]. The absence of a bleb in LOT reduces the likelihood of vision-threatening complications, such as a flat anterior chamber (AC), bleb leaks/infections, hypotony maculopathy, and choroidal detachment. These can develop following trabeculectomy in which antifibrotic agents are used [1,4].

The ab externo approach has been used to perform LOT in combination with metal trabeculotomes that incise a third of the meshwork [1,2,3], or with 5-0 and 6-0 polypropylene sutures, and a microcatheter that incises the full 360 degrees of the meshwork [5,6]. Surgeons have also reported using ab interno approaches with LOT techniques [7,8]. In 2015, we treated both eyes of one patient with steroid-induced glaucoma with a novel ab interno LOT procedure, which we referred to as microhook trabeculotomy (µLOT) [9]. As a result of the substantial reduction in IOP and less ocular surface invasiveness, we performed the procedure in other cases and reported our early postoperative results and safety profile in an initial case series [10,11]. We achieved a 43% IOP decrease, from the preoperative value of 25.9 to 14.7 mmHg postoperatively, with µLOT alone during the final 6 month evaluation [10]; when µLOT was combined with cataract surgery, we achieved a 28% decrease, i.e., from 16.4 to 11.8 mmHg postoperatively, at the final 9.5 month examination [11]. In the current study, we report the midterm surgical results and safety profile of µLOT in 560 consecutive eyes; the study included all cases in which µLOT was performed after the first case [9].

## 2. Materials and Methods

### 2.1. Methods

This retrospective observational case series included 560 consecutive glaucomatous eyes of 375 Japanese subjects (181 men, 194 women; mean age ± standard deviation (SD), 76.0 ± 13.2 years) who underwent µLOT performed by one surgeon (M.T.) at Matsue Red Cross Hospital between May 2015 and March 2018 to control the IOP. Preoperatively, the possible risks and benefits of µLOT, cataract surgery, and other possible glaucoma surgeries were explained to the patients, and the patients who chose µLOT alone or combined µLOT and cataract surgery underwent surgery. The study adhered to the tenets of the Declaration of Helsinki; the institutional review board (IRB) of Matsue Red Cross Hospital reviewed and approved the research (IRB No. 261). Preoperatively, all subjects provided written informed consent for surgery and use of the clinical data regarding the glaucoma treatment obtained during the follow-up periods. The patients’ demographic data and surgical procedures are summarized in Table 1. The mean follow-up was 405 days in this dataset.

### 2.2. Surgical Procedure

µLOT was performed as described previously [10,11]. Three specifically designed microhooks for µLOT, i.e., straight (M-2215S), right-angled (M-2215R), and left-angled (M-2215L) (all from Inami & Co., Ltd., Tokyo, Japan), were used [12]. When the combined procedure was performed, phacoemulsification cataract surgery was performed before µLOT; the cataract surgery was performed through a 2.2 mm wide clear corneal incision created at the 9 to 10 o’clock position (i.e., temporal incision for the right eye and nasal incision for the left eye) and a corneal port created at the 2 to 3 o’clock position. A one-piece soft-acrylic intraocular lens (IOL) was inserted through the same clear corneal incision; the Vivinex iSert XY1 IOL (Hoya, Tokyo, Japan) was used in most cases, and the AcrySof IQ IOL (Alcon Japan, Tokyo, Japan) and Tecnis OptiBlue IOL (AMO Japan, Tokyo, Japan) in others. After IOL implantation, standard sub-Tenon anesthesia was induced using 2% lidocaine (in most earlier cases) or intracameral anesthesia using 1% lidocaine (in most later cases). A viscoelastic material (1% sodium hyaluronate, Opegan Hi, Santen Pharmaceutical, Osaka, Japan) was also injected into the AC to widen the angle. Using a Swan-Jacob gonioprism lens (Ocular Instruments, Bellevue, WA, USA) to observe the angle, a microhook was inserted into the AC through the corneal incision. The tip of the microhook was then inserted into the Schlemm’s canal and moved circumferentially to incise the inner wall of Schlemm’s canal and TM over 3 clock hours. Using the same procedure, LOT was performed in the opposite angle using a microhook that was inserted through the corneal port. To improve the operability in most cases, a straight hook was used to incise the nasal angle and the right-angled and left-angled hooks were used to incise the temporal angle. After the viscoelastic material was aspirated, the corneal incision and port were closed by corneal stromal hydration. At the end of surgery, 1.65 mg of dexamethasone sodium phosphate (Decadron, Aspen, Japan, Tokyo, Japan) was injected subconjunctivally and 0.3% ofloxacin ointment (Tarivid, Santen Pharmaceutical) was applied. Finally, 1.5% levofloxacin (Nipro, Osaka, Japan) and 0.1% betamethasone (Sanbetason, Santen Pharmaceutical) were applied topically four times daily for 3 to 4 weeks (i.e., 1 bottle/eye), postoperatively in all cases. Topical non-steroidal anti-inflammatory drugs were not used routinely.

### 2.3. Measurements

The clinical parameters, including age, sex, glaucoma type, lens status, ocular surgical history, surgical procedure (i.e., µLOT alone or combined µLOT and cataract surgery), preoperative and postoperative best-corrected visual acuities (BCVA), IOP, number of antiglaucoma medications, AC flare measured using the FM-600 laser flare meter (Kowa, Nagoya, Japan), corneal endothelial cell density (CECD) measured using the EM-3000 specular microscope (Tomey, Nagoya, Japan), visual field mean deviation (MD) (central 30-2 program, Humphrey Visual Field Analyzer, Carl Zeiss Meditec, Dublin, CA, USA), and duration of postoperative follow-up were collected from the medical charts. The decimal BCVA was converted to the logarithm of the minimum angle of resolution VA. Counting fingers, hand motions, light perception, and no light perception were regarded as decimal VAs of 0.0025, 0.002, 0.0016, and 0.0013, respectively [13]. The IOP was measured using Goldmann applanation tonometry except for that measured on postoperative day 3 using the iCARE rebound tonometer (M.E. Technica, Tokyo, Japan). The site at which the LOT procedure was performed (i.e., nasal or temporal angle or both) and the extent, perioperative complications, simultaneous procedures other than regular cataract surgery, interventions for complications, and additional glaucoma surgeries performed were recorded by reviewing the medical and surgical records.

### 2.4. Statistical Analysis

The preoperative and final IOPs, medications, BCVA, AC flare, MD, and CECD were compared using the paired t-test. Successful IOP control was assessed by the fixed-point analysis and the survival curve analysis. For the fixed-point analysis, success was defined as a postoperative IOP of 18 mmHg or less and an IOP reduction of 20% or more compared with the preoperative value, or a postoperative IOP of 15 mmHg or less and IOP reduction of 20% or more compared with the preoperative value at the final visit. For survival curve analysis, the uncensored date was defined as the postoperative period of later than 90 days and the day when the IOP exceeded 18 mmHg (definitions A and C) or 15 mmHg (definitions B and D), IOP reductions of less than 20% (definitions A and B) or those that exceeded the baseline IOP (definitions C and D) with use of antiglaucoma medications, additional glaucoma surgery (all definitions), or loss of light perception (all definitions); the cases other than those that were uncensored were regarded as censored cases at the final visit. The cumulative incidence of additional glaucoma surgery after µLOT was analyzed by survival curve analysis; the uncensored date was defined as the day additional glaucoma surgery was performed, and the cases other than uncensored cases were regarded as censored cases at the final visit. To adjust for possible biases derived from the inclusion of both eyes of a patient and for differences in follow-up periods, the preoperative IOP and IOPs measured on day 3, weeks 1 to 2, and months 1 (3–5 weeks), 3 (2–4 months), 6 (5–7 months), 9 (8–10 months), 12 (11–14 months), 18 (15–21 months), 24 (22–27 months), 30 (28–33 months), and 36 (34–39 months) were compared using a mixed-effects regression model in which each patient’s identification number was regarded as a random effect and the time period as a fixed effect; this was followed by the t-test for the post-hoc comparison between groups. Postoperative changes in the number of antiglaucoma medications, BCVA, AC flare, MD, and CECD were also assessed using the mixed-effects regression model. In addition to the analyses in all eyes, the analyses were performed separately in eyes treated with µLOT alone or combined µLOT and cataract surgery separately. To assess the possible factors affecting the postoperative IOP, multiple regression analyses were performed; for the analyses, the IOP recorded at the final visit was a dependent variable, and age, gender, glaucoma types, surgical procedure, preoperative lens status, LOT extent, preoperative IOP, preoperative number of medications, and presence of postoperative complications were independent variables. All continuous data were expressed as the mean ± SD. All statistical analyses were performed using the JMP version 11.0 statistical software (SAS Institute, Inc., Cary, NC, USA). *p* < 0.05 was considered significant.

## 3. Results

Table 1 summarizes the patient data. Primary open-angle glaucoma (POAG) (57%) was the most frequent glaucoma type in this case series, followed by exfoliation glaucoma (20%), primary angle-closure glaucoma (PACG) including mixed-mechanism glaucoma (13%), steroid-induced glaucoma (3%), developmental glaucoma (3%), and others including secondary glaucoma due to uveitis or various causes (4%). µLOT was performed as an initial ocular surgery in 428 (76%) eyes. Among the 79 (14%) eyes with a history of previous cataract surgery, 47 eyes (8%) had no history of glaucoma surgery; thus, µLOT was performed as an initial glaucoma surgery in 475 (85%) eyes. µLOT was performed as a solo procedure in 159 (28%) eyes and combined procedure in 401 (72%) eyes; half of the eyes treated with the solo procedure were pseudophakic. µLOT was performed on both the nasal and temporal sides in 512 (92%) eyes, only on the nasal side in 24 (4%) eyes, and only on the temporal side in 24 (4%) eyes. The LOT extent was 6.9 h when µLOT was performed on both sides and 3.8 h and 3.6 h, respectively, when µLOT was performed only on the nasal or the temporal side. The duration of the follow-up was 405 ± 327 (range, 2–1326) days.

With the mixed-effects regression model, the postoperative changes in IOP were significant in the entire dataset, and in eyes treated with µLOT alone or combined µLOT (*p* < 0.0001 for each model) (Table 2). Compared with the preoperative data, in all eyes and eyes treated with µLOT alone or the combined procedure, the IOP reductions were significant (*p* < 0.0001–0.0072) at every time point up to 36 months postoperatively; the reductions in IOPs were 6.3 (31%) mmHg, 6.9 (31%) mmHg, and 6.0 (31%) mmHg in each group at the final visit, respectively. The postoperative changes in the number of antiglaucoma medications were significant in the entire dataset and in eyes treated with µLOT alone or combined µLOT (*p* < 0.0001 for each model) (Table 3). Compared with preoperatively, in the total dataset, the reductions in the number of antiglaucoma medications were significant up to 24 months (*p <* 0.0001–0.0191), but were not significant at 20 months and later, for up to 36 months postoperatively (*p* = 0.0918–0.2612). In all the eyes and eyes treated with µLOT alone or the combined procedure, the respective reductions in medications were 0.3 (11%), 0.5 (15%), and 0.3 (11%) in each group at the final visit; excluding 12 eyes for which data were missing, 534 (97%) eyes used at least one antiglaucoma medication at the final visit.

**Figure 1:**
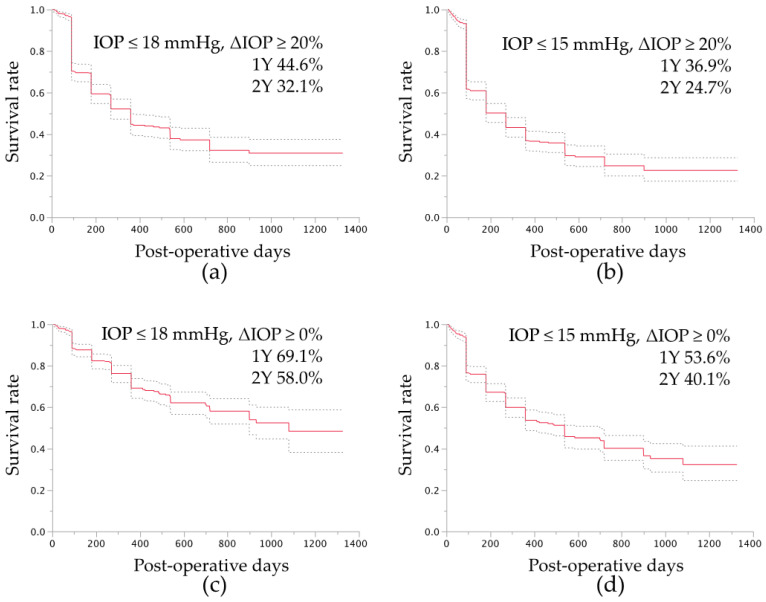
Success rate of intraocular pressure (IOP) control after microhook trabeculotomy by survival curve analysis. For the survival curve analysis, the uncensored date was defined as the postoperative period of later than 90 days and the day when the IOP level exceeded 18 mmHg (**a**,**c**) or 15 mmHg (**b**,**d**), IOP reduction of less than 20% (**a**,**b**), or exceeding the baseline IOP (**c**,**d**) with antiglaucoma medication, additional glaucoma surgery (**a**–**d**), or loss of light perception (**a**–**d**); the cases other than those that were uncensored were regarded as censored cases at the final visit. The dotted lines indicate the ranges of the 95% confidence intervals of the survival analysis. Y, years.

At the final visit, as assessed by the fixed-point success rate analysis, 379 (69%) eyes achieved successful IOP control of 18 mmHg or less and IOP reductions of 20% or more, and 349 (64%) eyes achieved successful IOP control of 15 mmHg or less and 20% IOP reductions of 20% or more. By life-table analysis, with antiglaucoma medication use, the success rates of IOP control of 18 mmHg or lower and IOP reductions of 20% or more, were 44.6% and 32.1% at postoperative years 1 and 2, respectively (Figure 1a), and the rates of 15 mmHg or lower and IOP reductions of 20% or more were 36.9% and 24.7% at postoperative years 1 and 2, respectively (Figure 1b). With the less demanding definitions of success, with antiglaucoma medication use, the success rates of IOP control of 18 mmHg or less and not exceeding the preoperative IOP were 69.1% and 58.0% at postoperative years 1 and 2, respectively (Figure 1c), and the rates of 15 mmHg or lower and not exceeding the preoperative IOP were 53.6% and 40.1% at postoperative years 1 and 2, respectively (Figure 1d).

**Figure 2:**
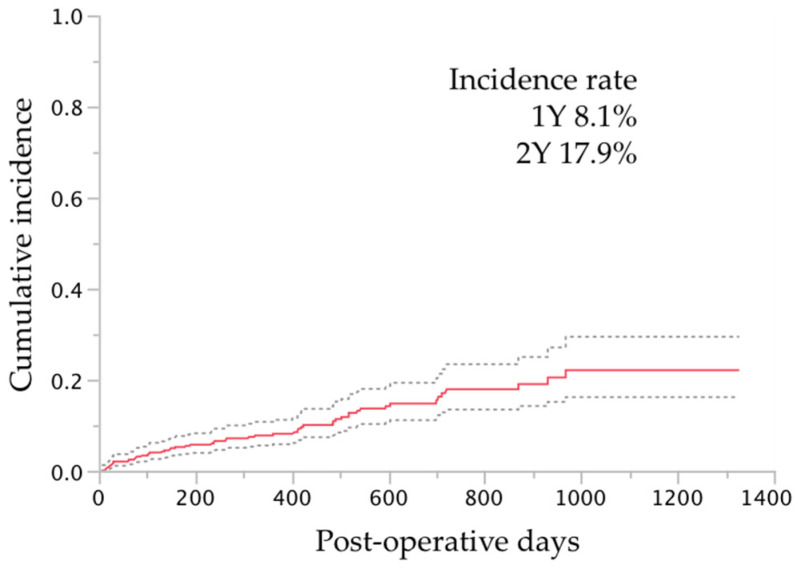
The cumulative incidence of additional glaucoma surgery after microhook trabeculotomy by survival curve analysis. For the survival curve analysis, the uncensored date was defined as the day when additional glaucoma surgery was performed; the cases other than those that were uncensored were regarded as censored at the final visit. The dotted lines indicate the ranges of the 95% confidence intervals of the survival analysis. Y, years.

Intraoperative complications and additional procedures were recorded in 24 (4%) eyes and 36 (6%) eyes, respectively (Table 4); most complications and additional procedures were related to the cataract surgery. The postoperative complications developed and interventions required were in 239 (43%) eyes and 63 (11%) eyes, respectively (Table 5). The most common postoperative complications and interventions other than additional glaucoma surgery were layered hyphema in 172 (30%) eyes and hyphema washout in 26 (5%), respectively. Additional glaucoma surgery was required in 57 (10%) eyes; the procedures included Ahmed Glaucoma Valve implantation in 21 (3%) eyes, trabeculectomy in 18 (3%) eyes, Ex-PRESS shunt in 13 (2%) eyes, µLOT in four (<1%) eyes, and goniosynechialysis and selective laser trabeculoplasty in one (<1%) eye each. The cumulative incidence rates of additional glaucoma surgery are shown in Figure 2. Additional surgeries were performed at 303 ± 264 days (range, 8–968 days) after the µLOT procedure.

In all eyes, compared with preoperatively, significantly better BCVA values (Table 6), higher AC flare values (Table 7), better visual field MD (Table 8), and fewer CECD (Table 9) were observed at the final visit (*p* < 0.0001–0.0011); these significant differences were observed in the combined surgery group (*p* < 0.0001–0.0004) but not in the µLOT-alone group (*p* = 0.1568–0.9069).

Finally, the possible factors associated with the postoperative IOP were assessed by multiple regression analyses (Table 10). Among the factors included in the model, older age, steroid-induced glaucoma, developmental glaucoma, and absence of postoperative complications were associated with lower final IOPs, and exfoliation glaucoma, other types of glaucoma (most cases were uveitic glaucoma with various etiology), and higher preoperative IOP were associated with higher final IOPs. Gender, solo or combined surgery, lens status, extent of trabeculotomy, and number of preoperative medications were not detected as a significant factor.

## 4. Discussion

This study included all 560 eyes treated with µLOT between the time when the procedure was developed in 2015 and March 2018. In the current cases, marked IOP reductions were achieved after the LOT procedure during the early to midterm postoperative period for up to 3 years in eyes with various glaucoma types. This agrees with the previous results after ab externo 120-degree LOT for POAG [1,2,3,14], exfoliation glaucoma [14,15], and PACG [16]. In eyes with POAG, the respective postoperative IOP and percentages of IOP reduction were, respectively, 15.4 mmHg and 13% 17 months after cataract surgery alone was performed [17], 16.1 mmHg and 24% 12 months after the ab externo 120-degree LOT procedure combined with cataract surgery [2,3], and 14.1 mmHg and 41% 12 months after phacotrabeculectomy in which mitomycin C was used [18]. Therefore, µLOT with/without cataract surgery achieved a 31% postoperative IOP reduction with medication at the final visit, comparable to or exceeding the reductions achieved with cataract surgery alone and ab externo 120-degree LOT combined with cataract surgery [2,3], and was less effective than phacotrabeculectomy with mitomycin C. However, this difference should be clarified in a comparative study. With medication use, about two-thirds of the current eyes achieved successful IOP control below 15 mmHg at the final visit by fixed-point analysis, and half of the cases achieved 15 mmHg 1 year postoperatively by life-table analyses (Figure 1d); thus, µLOT seems effective for normalizing the IOP at least during the early postoperative period, but its effect was not sufficiently powerful in cases that required target IOPs lower than normal or in cases in which medications had to stop.

In the current study, the surgeon determined the site at which LOT was performed, i.e., either in the temporal, nasal, or both angles, although in most (92%) cases LOT was performed in both angles. A perfusion study of autopsy eyes reported that incisions in the TM for 1, 4, and 12 clock hours eliminated 30%, 44%, and 51%, respectively, of outflow resistance, at a perfusion pressure of 7 mmHg, and 30%, 56%, and 72%, respectively, of outflow resistance at a perfusion pressure of 25 mmHg [19], indicating that different extents of LOT can result in different degrees of IOP reduction. Accordingly, it would be interesting to compare between IOP reductions with angle incisions on both sides and one side, and the correlations between the extent of the incisions and IOP reductions after µLOT. The current multivariate analyses showed that the extent of LOT (range, 2–10 clock hours) was not associated with the final postoperative IOP (Table 10). Previously, neither the optical coherence tomography (OCT)-detected extent of LOT opening after Trabectome (range; 0–160 degrees) [20], nor the extent of LOT during suture LOT (S-LOT) (range, 150–320 degrees) [21], was associated with the postoperative IOP. Other studies have reported greater IOP reductions with goniotomy performed using a Kahook Dual Blade (KDB) (extent of about 90 degrees) than with single iStent trabecular bypass implantation (lumen, 120 µm) [22,23,24]. Evidence suggests that goniotomy exceeding one quadrant might exert a clinically detectable maximal IOP reduction, but the possible correlation between the extent of µLOT and its efficacy is inconclusive and should be investigated further.

Various complications developed perioperatively (Table 4 and Table 5), although most resolved spontaneously or were treated with relatively minor interventions, such as washout of the hyphema. Macular edema (ME) seen on OCT has been reported to range from 4% to 11% after modern cataract surgery [25], and 4.3% after trabeculectomy [26]. In the Ahmed Baerveldt Comparison Study, cystoid ME was reported in 10 (3.6%) of 276 eyes within 3 months postoperatively [27], and in 13 (4.7%) eyes after 3 months for up to 5 years postoperatively [28]. Accordingly, the incidence of ME (22 eyes, 4%) in this case series was equivalent to that of cataract surgery or filtration surgeries. The absence of the use of topical non-steroidal anti-inflammatory drugs may be associated with the incidence of ME, although the association was undetermined. In cases with combined surgery, no severe complications associated with cataract surgery developed, and the VA (Table 6) or visual field (Table 8) improved significantly at the final visit compared to the preoperative values. Thus, simultaneous cataract surgery with µLOT resulted in visual function recovery in eyes with glaucoma with visually relevant cataracts. A transient IOP spike was reported in 15.2% to 29% after ab externo LOT [2,15,29,30], 28% to 33% after S-LOT [31,32], 5.4% after Trabectome [33], and 5.7% after KDB [34]. Thus, the incidence of an IOP spike after µLOT (6%) seems lower than ab externo LOT or ab interno LOT with a wider incision, and equivalent to other ab interno goniotomy procedures. In advanced cases, a postoperative IOP spike is potentially vision-threatening. Although some surgeons have reported the clinical relevance of performing Trabectome for advanced glaucoma [35], we recommend that the decision to perform µLOT should be considered carefully in glaucomatous eyes with advanced visual field defects. We observed increased AC flare after µLOT (Table 7), which returned to the preoperative level by 6 months postoperatively. As reported previously, postoperative increases in AC flare after µLOT might last longer than filtration surgery, such as Ex-PRESS shunt [36]. The loss of CECD was reported to be 6.5% 1 year after cataract surgery alone in eyes with glaucoma [37], 6.3% 2 years after trabeculectomy monotherapy [38], and 2.4% 1 year after Trabectome (combined surgery, 47%) [39]. In our cases, the rates of losses of CECD were 0% and 6% after monotherapy and combined surgery, respectively (Table 9); thus, µLOT itself seems to have minimal impact on the surgical loss of CECD. In the current case series, persistent hypotony subsequent to ciliochoroidal detachment (CCD) developed in four eyes. As discussed previously [40], increased uveoscleral outflow due to LOT [41] or the creation of a cyclodialysis cleft by traction of the pectinate ligament [40] can be a mechanism of CCD development. Akagi et al. reported that a CCD detected by anterior-segment OCT developed in 14 (42%) of 33 eyes 3 days after a Trabectome procedure; the CCD persisted in four eyes (12%) at 1 month and resolved by 3 months [42]. Sato et al. reported that CCDs detected by anterior-segment OCT developed in 21 (48%) of 44 eyes within 7 days after S-LOT; the CCDs resolved in 19 eyes within 1 month and in two eyes by 3 months [43]. Cyclodialysis and hypotony maculopathy were reported in one case after KDB [44]. Thus, CCD itself is not rare after goniotomy/LOT, and although rare, hypotony might persist after ab interno goniotomy/LOT.

Intraoperatively, the incisional depth can be controlled by monitoring the tip of the hook through the TM, based on the resistance. This allows surgeons to make a selective incision of the TM/inner wall of the Schlemm’s canal with minimal damage to the outer wall of the Schlemm’s canal; incising the inner wall without damaging the outer wall of the Schlemm’s canal may be difficult when using a straight knife. µLOT seems to be an easier procedure than traditional goniotomy. Conjunctival and scleral sparing with the ab interno technique, short surgical time, moderate IOP reduction, and no bleb-related complications fulfill the conditions of minimally invasive glaucoma surgery [45,46], as with the recent techniques of ab interno LOT/trabeculectomy and gonio-bypass surgeries, such as the Trabectome [33], iStent [47], gonioscopy-assisted transluminal LOT/S-LOT [7,8], ab interno canaloplasty [48], and KDB [49,50]. Because of the minimally invasive characteristics of the surgery, the µLOT can be performed at the time of the surgery for visually significant cataracts in glaucoma eyes, and this can explain the inclusion of eyes with low preoperative IOP in this dataset. The low surgical cost because of no requirement for expensive devices and the use of reusable hooks are other advantages of our procedure; thus, µLOT can be a suitable procedure, especially in areas/countries with resource-poor settings.

The limitations of the current study included the absence of a control group, the retrospective design, the short mean follow-up, and the inclusion of eyes with various glaucoma types and previous ocular surgeries. Large numbers of lost follow-up are another limitation for the implication of surgical efficacy in this study; however, we believe that including all 560 eyes into the analyses might have merit to provide unbiased information regarding the adverse events of this surgical procedure. Based on the multivariate analyses (Table 10), among the glaucoma types, steroid-induced glaucoma and developmental glaucoma are especially good candidates for µLOT, which agreed with previous studies of ab externo LOT [51,52]. The inclusion of both eyes of a patient, various follow-up periods, and missing data may also have introduced bias, although we minimized this by using a mixed-effects regression model. We believe that the current results show that µLOT is worth further evaluation in a comparative study of other surgeries, such as cataract surgery alone or other TM surgeries.

## 5. Conclusions

In summary, the mean preoperative intraocular pressure (IOP) (20.2 mmHg) and number of antiglaucoma medications (2.8) decreased 31% (13.9 mmHg) and 11% (2.5), respectively, at the mean final visit of 405 days postoperatively. In conclusion, µLOT has a significant IOP-lowering potential in patients with glaucoma, and improves visual function when combined with cataract surgery.

## Figures and Tables

**Table 1:** Demographic Patient Data

Parameters	Mean ± SD (Range) or No. (%)
Eyes/Subjects	560/375
By subjects	
Age, years	70.6 ± 13.2 (11, 95)
Sex, subjects (%)	
Male	181 (48)
Female	194 (52)
By eyes	
Glaucoma type, eyes (%)	
POAG	317 (57)
EXG	112 (20)
PACG/mixed	71 (13)
Steroid	21 (3)
Developmental	17 (3)
Others	22 (4)
No. previous ocular surgeries	0.3 (0–3)
Previous ocular surgeries, eyes (%)	
No	428 (76)
Yes	132 (24)
Cataract surgery	79 (14)
Phacoemulsification	79 (14)
Glaucoma surgery	73 (13)
Trabeculectomy	4 (<1)
Ex-PRESS shunt	7 (1)
ab externo trabeculotomy	27 (5)
Goniocynechialysis	12 (2)
Gold shunt	1 (<1)
Laser iridotomy	14 (3)
Selective laser trabeculoplasty	13 (2)
Laser trabeculoplasty	1 (<1)
Laser gonioplasty	2 (<1)
Retinal surgery	13 (2)
Retinal photocoagulation	7 (1)
Scleral buckling	4 (<1)
Pars plana vitrectomy	2 (<1)
Surgical procedure, eyes (%)	
µLOT alone	159 (28)
Phakic eye	80 (14)
Pseudophakic eye	79 (14)
µLOT + cataract surgery	401 (72)
Trabeculotomy site, eyes (%)	
Nasal and temporal	512 (92)
Nasal only	24 (4)
Temporal only	24 (4)
Extent of trabeculotomies (clock hours)	
Nasal and temporal	6.9 ± 0.9 (4, 10)
Nasal only	3.8 ± 0.4 (3, 4)
Temporal only	3.6 ± 0.7 (2, 5)
Follow-up, days	
Mean ± SD (range)	405 ± 327 (2, 1326)

Abbreviations: POAG, primary open-angle glaucoma; EXG, exfoliation glaucoma; PACG, primary angle-closure glaucoma; Mixed, mixed mechanism glaucoma; Steroid, steroid-induced glaucoma; Developmental, developmental glaucoma; µLOT, microhook ab interno trabeculotomy; SD, standard deviation.

**Table 2:** Preoperative and Postoperative Intraocular Pressures (mmHg) Compared by Mixed-Effects Regression Model.

Periods	Total			µLOT Alone			µLOT + Cataract Surgery		
	No. Eyes	Mean ± SD (Range)	*p* Value ‡	No. Eyes	Mean ± SD (Range)	*p* Value ‡	No. Eyes	Mean ± SD (Range)	*p* Value ‡
Preoperative	560	20.2 ± 7.0 (10, 69)		159	22.4 ± 8.6 (12, 69)		401	19.3 ± 6.0 (10, 63)	
3 days	547	9.7 ± 5.2 (1, 51)	<0.0001	154	10.9 ± 5.7 (4, 44)	<0.0001	393	9.3 ± 5.0 (1, 51)	<0.0001
1–2 weeks	546	14.1 ± 6.1 (1, 46)	<0.0001	155	16.0 ± 7.0 (3, 42)	<0.0001	391	13.4 ± 5.5 (1, 46)	<0.0001
1 month	506	13.5 ± 5.3 (0, 59)	<0.0001	142	14.8 ± 5.5 (0, 40)	<0.0001	364	13.0 ± 5.2 (1, 59)	<0.0001
3 months	431	12.9 ± 3.5 (1, 30)	<0.0001	118	14.4 ± 4.1 (1, 30)	<0.0001	313	12.3 ± 3.0 (3, 25)	<0.0001
6 months	369	13.0 ± 3.5 (3, 29)	<0.0001	99	14.4 ± 3.7 (7, 29)	<0.0001	270	12.4 ± 3.3 (3, 29)	<0.0001
9 months	311	13.1 ± 3.6 (4, 32)	<0.0001	86	14.6 ± 3.6 (7, 32)	<0.0001	225	12.5 ± 3.4 (4, 23)	<0.0001
12 months	265	13.2 ± 4.2 (4, 51)	<0.0001	64	14.4 ± 3.0 (8, 21)	<0.0001	201	12.8 ± 4.5 (4, 51)	<0.0001
18 months	204	12.9 ± 3.6 (4, 27)	<0.0001	53	14.4 ± 3.7 (7, 23)	<0.0001	151	12.3 ± 3.5 (4, 27)	<0.0001
24 months	133	12.8 ± 3.7 (5, 38)	<0.0001	37	13.9 ± 3.2 (9, 20)	<0.0001	96	12.4 ± 3.8 5, 38)	<0.0001
30 months	74	12.8 ± 3.6 (6, 25)	<0.0001	21	13.2 ± 4.0 (6, 19)	0.0005	53	12.6 ± 3.4 (8, 25)	<0.0001
36 months	47	13.3 ± 3.3 (7, 25)	<0.0001	12	14.9 ± 3.9 (11, 25)	0.0072	35	12.7 ± 2.8 (7, 24)	<0.0001
Final visit	549	13.9 ± 5.6 (4, 59)	<0.0001	157	15.5 ± 6.3 (6, 41)	<0.0001	392	13.3 ± 5.2 (4, 59)	<0.0001
		*p* < 0.0001 †			*p* < 0.0001 †			*p* < 0.0001 †	

Data are expressed as means ± standard deviations (range). † *p* values are calculated using the mixed-effects regression model. ‡ *p* values are calculated using the paired *t*-test between preoperative and respective time period values. Abbreviations: µLOT, microhook trabeculotomy; SD, standard deviation.

**Table 3:** Preoperative and Postoperative Medications Compared by Mixed-Effects Regression Model.

Periods	Total			µLOT Alone			µLOT + Cataract Surgery		
	No. Eyes	Mean ± SD (Range)	*p* Value ‡	No. Eyes	Mean ± SD (Range)	*p* Value ‡	No. Eyes	Mean ± SD (Range)	*p* Value ‡
Preoperative	560	2.8 ± 1.1 (0, 6)		159	3.3 ± 1.1 (0, 6)		401	2.7 ± 1.1 (0, 5)	
3 days	546	2.4 ± 1.0 (0, 5)	<0.0001	152	2.8 ± 0.9 (0, 4)	<0.0001	394	2.3 ± 1.0 (0, 5)	<0.0001
1–2 weeks	544	2.4 ± 0.9 (0, 4)	<0.0001	155	2.7 ± 0.8 (0, 4)	<0.0001	389	2.3 ± 1.0 (0, 4)	<0.0001
1 month	497	2.5 ± 0.9 (0, 5)	<0.0001	137	2.7 ± 0.9 (0, 5)	<0.0001	360	2.4 ± 0.9 (0, 4)	<0.0001
3 months	427	2.4 ± 0.9 (0, 4)	<0.0001	116	2.7 ± 0.9 (0, 4)	<0.0001	311	2.3 ± 0.9 (0, 4)	<0.0001
6 months	362	2.4 ± 1.0 (0, 5)	<0.0001	96	2.8 ± 0.9 (0, 5)	<0.0001	266	2.3 ± 0.9 (0, 4)	<0.0001
9 months	303	2.4 ± 0.9 (0, 4)	<0.0001	85	2.8 ± 0.8 (1, 4)	<0.0001	218	2.3 ± 0.9 (0, 4)	<0.0001
12 months	263	2.5 ± 0.9 (0, 4)	<0.0001	65	2.8 ± 0.8 (1, 4)	0.0007	198	2.4 ± 0.9 (0, 4)	0.0004
18 months	200	2.6 ± 0.9 (0, 4)	0.0052	53	2.9 ± 0.8 (1, 4)	0.0053	147	2.5 ± 0.9 (0, 4)	0.0472
24 months	127	2.5 ± 1.0 (0, 4)	0.0191	37	2.9 ± 0.8 (1, 4)	0.0007	90	2.3 ± 1.0 (0, 4)	0.5559
30 months	74	2.5 ± 0.9 (1, 4)	0.0918	21	2.7 ± 1.0 (1, 4)	0.0014	53	2.4 ± 0.9 (1, 4)	1.0000
36 months	47	2.4 ± 1.0 (1, 4)	0.2612	12	2.6 ± 1.2 (1, 4)	0.0069	35	2.4 ± 0.9 (1, 4)	0.6378
Final visit	548	2.5 ± 1.0 (0, 5)	<0.0001	157	2.8 ± 0.9 (0, 5)	<0.0001	391	2.4 ± 1.0 (0, 5)	<0.0001
		*p* < 0.0001 †			*p* < 0.0001 †			*p* < 0.0001 †	

Data are expressed as means ± standard deviations (range). † *p* values are calculated using the mixed-effects regression model. ‡ *p* values are calculated using the paired *t*-test between preoperative and respective time period values. Abbreviations: µLOT, microhook trabeculotomy; SD, standard deviation.

**Table 4:** Intraoperative Complications and Interventions

Complications, *n* (%)		Interventions, *n* (%)	
Iris prolapse, IFIS	15 (3)	CTR implantation	13 (2)
Angle recession	4 (<1)	Sub-Tenon triamcinolone injection	9 (2)
Posterior capsule rapture	3 (<1)	Goniocynechialysis	7 (1)
Unable to observe angle due to	2 (<1)	Pupillary sphincterotomy	6 (1)
Bleeding from angle	1(<1)	Anterior vitrectomy	2 (<1)
Exotropia	1(<1)	Hyphema washout	1 (<)
		ICL removal	1 (<)
		Synechialysis of Ex-PRESS shunt	1 (<)
Any complication	24 (4)	Any intervention	36 (6)

Abbreviations: IFIS, intraoperative floppy iris syndrome; CTR, capsular tension ring; ICL, implantable collamer lens.

**Table 5:** Postoperative Complications and Interventions

Complications, *n* (%)		Interventions, *n* (%)	
Layered hyphema	172 (30)	Hyphema washout	26 (5)
Transient IOP elevation >30 mmHg	34 (6)	Anterior chamber injection of tPA	15 (3)
Fibrin formation in anterior chamber	24 (4)	Posterior synechialysis, pupiloplasty	8 (1)
Macular edema	22 (4)	Cataract surgery	6 (1)
Posterior synechia, corectopia, pupillary occlusion	9 (2)	combined with glaucoma surgery	5 (<1)
cataract	6 (1)	Sub-Tenon triamcinolone injection	4 (<1)
Vitreous hemorrhage	5 (<1)	Pars-plana vitrectomy	3 (<1)
Blood accumulation in the lens bag	5 (<1)	Intravitreal anti-VEGF injection	3 (<1)
Persistent hypotony	4 (<1)	Nd:YAG laser capsulotomy	2 (<1)
Keratic precipitates, iritis	3 (<1)	Anterior chamber paracentesis	1 (<1)
After cataract	2 (<1)	Anterior chamber SF6 gas injection	1 (<1)
Contraction of CCC edge	1 (<1)	Anterior chamber OVD injection	1 (<1)
Choroidal hemorrhage	1 (<1)	Incision of CCC edge by Nd:YAG laser	1 (<1)
Branch retinal vein occlusion	1 (<1)		
Age-related macular degeneration	1 (<1)		
Any complications	239 (43)	Any intervention	63 (11)

Abbreviations: IOP, intraoperative pressure; IFIS, intraoperative floppy iris syndrome; CCC, continuous curvilinear capsulorrhexis; tPA, tissue plasminogen activator; VEGF, vascular endothelial growth factor; Nd:YAG, neodymium:yttrium–aluminum–garnet; SF6, sulfur hexafluoride; OVD, ocular viscoelastic device.

**Table 6:** Preoperative and Postoperative Best-Corrected Visual Acuity (Logarithm of the Minimum Angle of Resolution) Compared by Mixed-Effects Regression Model.

Periods	Total			µLOT Alone			µLOT + Cataract Surgery		
	No. Eyes	Mean ± SD (Range)	*p* Value ‡	No. Eyes	Mean ± SD (Range)	*p* Value ‡	No. Eyes	Mean ± SD (Range)	*p* Value ‡
Preoperative	560	0.23 ± 0.42 (−0.08, 2.80)		159	0.11 ± 0.39 (−0.08, 2.60)		401	0.28 ± 0.43 (−0.08, 2.80)	
1–2 weeks	538	0.33 ± 0.61 (−0.08, 2.89)	<0.0001	153	0.30 ± 0.68 (−0.08, 2.70)	<0.0001	385	0.34 ± 0.58 (−0.0,8, 2.89)	0.0442
1 month	494	0.19 ± 0.46 (−0.18, 2.89)	0.0262	136	0.15 ± 0.48 (−0.18, 2.80)	0.0180	358	0.21 ± 0.45 (−0.08, 2.89)	<0.0001
3 months	429	0.13 ± 0.37 (−0.08, 2.89)	<0.0001	116	0.07 ± 0.29 (−0.08, 2.00)	0.8300	313	0.15 ± 0.39 (−0.08, 2.89)	<0.0001
6 months	367	0.14 ± 0.40 (−0.18, 2.89)	<0.0001	98	0.10 ± 0.37 (−0.08, 2.60)	0.2943	269	0.15 ± 0.42 (−0.18, 2.89)	<0.0001
9 months	309	0.11 ± 0.42 (−0.18, 2.89)	<0.0001	86	0.04 ± 0.34 (−0.18, 2.70)	0.4275	223	0.13 ± 0.44 (−0.08, 2.89)	<0.0001
12 months	270	0.10 ± 0.39 (−0.18, 2.89)	<0.0001	66	0.06 ± 0.36 (−0.18, 2.60)	0.2679	204	0.12 ± 0.40 (−0.08, 2.89)	<0.0001
18 months	201	0.11 ± 0.45 (−0.08, 2.89)	<0.0001	53	0.03 ± 0.20 (−0.08, 1.10)	0.0790	148	0.14 ± 0.50 (−0.08, 2.89)	<0.0001
24 months	133	0.11 ± 0.41 (−0.18, 2.89)	0.0011	37	0.03 ± 0.24 (−0.08, 1.22)	0.0489	96	0.14 ± 0.46 (−0.18, 2.89)	0.0002
30 months	74	0.11 ± 0.47 (−0.08, 2.89)	0.0041	21	0.05 ± 0.28 (−0.08, 1.10)	0.1899	53	0.14 ± 0.53 (−0.08, 2.89)	0.0004
36 months	47	0.15 ± 0.57 (−0.18, 2.89)	0.0588	12	0.01 ± 0.14 (−0.08, 0.40)	0.6985	35	0.20 ± 0.66 (−0.08, 2.89)	0.0519
Final visit	547	0.14 ± 0.41 (−0.18, 2.89)	<0.0001	156	0.11 ± 0.38 (−0.18, 2.70)	0.2317	391	0.15 ± 0.42 (−0.18, 2.89)	<0.0001
		*p* < 0.0001 †			*p* < 0.0001 †			*p* < 0.0001 †	

Data are expressed as means ± standard deviations (range). † *p* values are calculated using the mixed-effects regression model. ‡ *p* values are calculated using the paired *t*-test between preoperative and respective time period values. Abbreviations: µLOT, microhook trabeculotomy; SD, standard deviation.

**Table 7:** Preoperative and Postoperative Anterior Chamber Flare (Photon Counts/msec) Compared by Mixed-Effects Regression Model.

Periods	Total			µLOT Alone			µLOT + Cataract Surgery		
	No. Eyes	Mean ± SD (Range)	*p* Value ‡	No. Eyes	Mean ± SD (Range)	*p* Value ‡	No. Eyes	Mean ± SD (Range)	*p* Value ‡
Preoperative	527	13.7 ± 13.3 (1.0, 141.3)		149	13.4 ± 14.5 (1, 111.3)		378	13.8 ± 12.8 (1, 141.3)	
1–2 weeks	472	69.8 ± 82.8 (3.6, 621.7)	<0.0001	130	53.1 ± 78.1 (3.6, 424.6)	<0.0001	342	76.2 ± 83.7 (5.1, 621.7)	<0.0001
1 month	421	27.1 ± 24.6 (3.6, 278.7)	<0.0001	120	16.8 ± 16.3 (3.6, 116.6)	0.1013	301	31.2 ± 26.1 (4.2, 278.7)	<0.0001
3 months	363	16.7 ± 13.2 (2.5, 152.7)	0.0035	101	11.6 ± 7.4 (3.1, 44.7)	0.2899	262	18.6 ± 14.4 (2.5, 152.7)	0.0001
6 months	314	14.0 ± 10.4 (3.3, 111.2)	0.3578	91	11.2 ± 12.0 (3.3, 111.2)	0.1460	223	15.2 ± 9.5 (4.8, 74)	0.1141
9 months	250	13.1 ± 8.5 (1.3, 56.6)	0.8366	77	9.8 ± 6.7 (3.8, 49.4)	0.0338	173	14.5 ± 8.9 (1.3, 56.6)	0.0469
12 months	218	13.2 ± 8.9 (3.4, 64.0)	0.9811	59	11.5 ± 8.6 (3.4, 44.1)	0.3022	159	13.8 ± 8.9 (4.6, 64.0)	0.3422
18 months	164	12.9 ± 9.0 (4.6, 92.1)	0.9159	49	12.3 ± 12.5 (4.9, 74.6)	0.9536	115	13.2 ± 7.1 (4.6, 49.5)	0.8654
24 months	85	12.3 ± 6.1 (4.5, 39.6)	0.4559	31	10.5 ± 4.4 (5.1, 24.7)	0.1305	54	13.2 ± 6.8 (4.5, 39.6)	0.4887
30 months	45	11.6 ± 4.9 (5.5, 30.4)	0.6339	17	10.5 ± 3.7 (6.9, 19.9)	0.4846	28	12.4 ± 5.4 (5.5, 30.4)	0.9692
36 months	22	11.3 ± 4.4 (6.2, 24.9)	0.3337	8	9.7 ± 3.4 (6.4, 17.2)	0.1545	14	12.1 ± 4.8 (6.2, 24.9)	0.7522
Final visit	494	17.8 ± 23.0 (3.6, 304.3)	0.0011	146	15.6 ± 26.9 (3.6, 61.4)	0.4406	348	18.8 ± 21.2 (4.2, 247.7)	0.0004
		*p* < 0.0001 †			*p* < 0.0001 †			*p* < 0.0001 †	

Data are expressed as means ± standard deviations (range). † *p* values are calculated using the mixed-effects regression model. ‡ *p* values are calculated using the paired *t*-test between preoperative and respective time period values. Abbreviations: µLOT, microhook trabeculotomy; SD, standard deviation.

**Table 8:** Preoperative and Postoperative Visual Field MD (dB) Compared by Mixed-Effects Regression Model.

Periods	Total			µLOT Alone			µLOT + Cataract Surgery		
	No. Eyes	Mean ± SD (Range)	*p* Value ‡	No. Eyes	Mean ± SD (Range)	*p* Value ‡	No. Eyes	Mean ± SD (Range)	*p* Value ‡
Preoperative	522	−12.5 ± 8.0 (−30.98, +1.55)		152	−12.3 ± 8.5 (−30.03, +0.75)		370	−12.6 ± 7.9 (−30.98, +1.55)	
12 months	237	−10.8 ± 8.2 (−31.82, +3.00)	<0.0001	61	−10.2 ± 7.8 (−30.44, +0.60)	0.1148	176	−11.0 ± 8.3 (−31.82, +3.00)	0.0005
24 months	115	−10.5 ± 8.1 (−29.80, +1.99)	0.0011	32	−12.3 ± 9.1 (−29.80, −0.89)	0.3891	83	−9.8 ± 7.7 (−29.39, +1.19)	0.0060
36 months	41	−7.83 ± 7.7 (−23.31, +1.33)	0.0588	11	−9.7 ± 7.7 (−22.35, −0.35)	0.7044	30	−7.14 ± 7.8 (−23.31, +1.33)	0.0005
Final visit	406	−11.9 ± 8.2 (−31.49, +1.33)	<0.0001	111	−12.8 ± 8.9 (−31.49, +0.60)	0.9069	295	−11.6 ± 7.9 (−30.35, +1.33)	<0.0001
		*p =* 0.0247 †			*p =* 0.6145 †			*p* = 0.0130 †	

Data are expressed as means ± standard deviations (range). † *p* values are calculated using the mixed-effects regression model. ‡ *p* values are calculated using the paired *t*-test between preoperative and respective time period values. Abbreviations: µLOT, microhook trabeculotomy; SD, standard deviation.

**Table 9:** Preoperative and Postoperative CECD (cells/mm2) Compared by Mixed-Effects Regression Model.

Periods	Total			µLOT Alone			µLOT + Cataract Surgery		
	No. Eyes	Mean ± SD (Range)	*p* Value ‡	No. Eyes	Mean ± SD (Range)	*p* Value ‡	No. Eyes	Mean ± SD (Range)	*p* Value ‡
Preoperative	547	2381 ± 374 (526, 3308)		152	2385 ± 405 (1118, 3043)		395	2379 ± 363 (526, 3143)	
12 months	323	2326 ± 371 (1188, 3174)	0.0006	91	2362 ± 419 (1188, 3043)	0.5871	232	2312 ± 350 (1223, 3174)	0.0001
24 months	224	2347 ± 369 (1167, 3481)	0.0006	60	2340 ± 410 (1167, 3037)	0.6857	164	2349 ± 354 (1246, 34,381)	0.0002
36 months	94	2267 ± 360 (1219, 2979)	0.0015	31	2227 ± 428 (1219, 2885)	0.4850	63	2287 ± 324 (1573, 2979)	0.0002
Final visit	26	2230 ± 472 (1065, 2875)	0.0517	8	2402 ± 452 (1421, 2875)	0.3839	18	2153 ± 472 (1065, 2782)	0.1162
	494	2246 ± 422 (523, 2875)	<0.0001	142	2279 ± 449 (1147, 3037)	0.1568	352	2233 ± 410 (523, 3174)	<0.0001

Data are expressed as means ± standard deviations (range). † *p* values are calculated using the mixed-effects regression model. ‡ *p* values are calculated using the paired *t*-test between preoperative and respective time period values. Abbreviations: µLOT, microhook trabeculotomy; SD, standard deviation.

**Table 10:** Assessment of Factors Associated with Postoperative Intraocular Pressure Levels.

Parameters	r (95% CI Range)	Standard β	*p* Value
Age (/year)	−0.08 (−0.13, −0.03)	−0.18	0.0015
Female (/male)	0.15 (−0.28, 0.60)	0.03	0.4869
Glaucoma type (/POAG)			0.0036
EXG	1.21 (0.06, 2.36)	0.17	0.0395
PACG (including Mixed)	0.16 (−1.11, 1.44)	0.02	0.8022
Steroid	−2.78 (−4.90, −0.65)	−0.28	0.0106
Development	−2.44 (−4.72, −0.15)	−0.24	0.0364
Others	2.94 (0.96, 4.92)	0.30	0.0037
µLOT alone (/combined µLOT + cataract surgery)	0.17 (−0.63, 0.97)	0.03	0.6794
Phakic eye (/pseudophakic eye)	−0.34 (−1.32, 0.64)	−0.04	0.4938
Extent of trabeculotomy (/clock hours)	0.10 (−0.27, 0.48)	0.02	0.5935
Preoperative IOP (/mmHg)	0.32 (0.25, 0.39)	0.39	<0.0001
Preoperative number of medications (/medication)	0.18 (−0.21, 0.58)	0.04	0.3613
Absence of postoperative complications (/presence of complication)	−0.52 (−0.96, −0.10)	−0.09	0.0159

Possible associations between IOP at final visit and various parameters are assessed using multiple regression analysis. Abbreviations: POAG, primary open-angle glaucoma; EXG, exfoliation glaucoma; PACG, primary angle-closure glaucoma; Mixed, mixed mechanism glaucoma; Steroid, steroid-induced glaucoma; Development, developmental glaucoma; µLOT, microhook ab interno trabeculotomy; r, regression coefficient; CI, confidence interval.

## Data Availability

Data is fully available upon reasonable request to corresponding author.
